# Evaluation of dentinal defect formation after root canal preparation with two reciprocating systems and hand instruments: an *in vitro* study

**DOI:** 10.1080/13102818.2014.996982

**Published:** 2015-01-13

**Authors:** Dilek Helvacioglu-Yigit, Seda Aydemir, Ayca Yilmaz

**Affiliations:** ^a^ Department of Endodontics, Faculty of Dentistry, Kocaeli University, Kocaeli, Turkey; ^b^ Department of Endodontics, Faculty of Dentistry, Istanbul University, Istanbul, Turkey

**Keywords:** reciprocating instruments, root canal preparations, vertical root fracture

## Abstract

The purpose of this study was to evaluate the presence of dentinal defects after root canal preparation with hand instruments and two different reciprocating instruments. Sixty freshly extracted mandibular incisor teeth were selected for this *in vitro* study. On the basis of root length, mesiodistal and buccolingual dimensions, the teeth were allocated into three identical experimental groups (*n* = 15) and one control group (*n* = 15). The teeth in the control group were left unprepared. The other groups were: stainless steel hand instruments, WaveOne® Primary instruments and RECIPROC® R25 instruments. The reciprocating instruments were used with a reciprocating gentle in-and-out motion in a torque-limited electric motor at the appropriate preset mode. Horizontal sections were made 3, 6 and 9 mm from the apex. Samples were stained with methylene blue and viewed through a stereomicroscope. The presence of dentinal defects (fractures, incomplete cracks and craze lines) and their locations were investigated by two endodontists. These data were analysed statistically by Fisher's exact and chi-square tests. No defects were observed in the unprepared group. All instruments caused dentinal defects, with no significant differences between the instrument systems. All experimental groups demonstrated significantly more defects at the 3-mm level in comparison with the unprepared group (*p* = 0.032). At the other levels, there was no significant difference between the experimental groups and the control group. The use of hand or reciprocating instruments could induce the formation of dentinal defects during root canal preparation.

## Introduction

Vertical root fracture (VRF) is a clinical complication that has a potential impact on treatment decisions. Various factors such as physical trauma, occlusal prematurity, repetitive heavy and stressful chewing, resorption-weakening and dental procedures have been found to be causative factors in the development of VRFs.[1–3] Coronal and radicular tooth structure loss predisposes endodontically treated teeth to fracture, due to prior pathology or endodontic and/or restorative treatment procedures.[4] Endodontic procedures might also influence fracture patterns as well as other defects, such as craze lines or partial cracks, which have the potential to lead to VRFs.[5]

Many factors – such as structural changes or loss of tooth substance [4] due to chemo-mechanical preparation [5–7] and intracanal dressings,[8] as well as obturation or restorative procedures during or after endodontic treatment – may lead to the development of VRFs. However, any single factor can ultimately be the trigger. Fracture susceptibility depends primarily on the final canal shape, the extent of canal enlargement and the elimination of irregularities, which are stress concentration sites.[9] Thus, different instrumentation techniques and systems, with different cutting blade and tip designs and tapers, lead to different types and degrees of dentinal damage to the root canal wall.[6,7,10]

The step-back technique using hand instruments differs from NiTi rotary instrumentation in terms of size, shape and taper of root canal preparation. This leads to three-dimensional canal forms with different characteristics after preparation.[11] Despite various clinical advantages, NiTi rotary systems have been shown to produce a higher incidence of dentinal defects than hand instrumentation.[6,7,12] Recently, two new reciprocating systems have been introduced: RECIPROC® (VDW, Munich, Germany) and WaveOne® (Dentsply Maillefer, Ballaigues, Switzerland). These instruments are manufactured with M-Wire NiTi alloy [13] and are recommended for use with a dedicated reciprocating motor in preset modes with different angles of rotation and speed. (RECIPROC® instruments use the RECIPROC ALL mode with 150° counterclockwise motion followed by 30° clockwise rotation with a speed of 300 rpm, whilst WaveOne® instruments use the WAVEONE ALL mode with 170° counterclockwise motion followed by 50° clockwise rotation with a speed of 350 rpm.[14] The superiority of the reciprocating motion is based on the ‘balanced force’ concept, which is explained by the ‘action and reaction’ law in physics.[15] The reciprocating movement results in minimized torsional and flexural stresses and a reduced binding effect of the instrument against dentine. It has also advantages of creating less-invasive root canal preparations by increasing canal centring.[16,17] However, there is limited knowledge about the clinical relevance of these advantages, and only a few articles about dentinal damage induced by reciprocating instruments.[18]

The purpose of this study was to evaluate the presence of dentinal defects after root canal preparation with hand instruments and two different reciprocating instruments.

## Materials and methods

### Selection and preparation of teeth

Two hundred and ten freshly extracted mandibular anterior teeth with single, straight roots and intact root apices were obtained from the collections of the Department of Oral and Maxillofacial Surgery, Kocaeli University. The teeth had been extracted for routine clinical reasons. Patients were informed that the extracted teeth were to be used for research purposes and provided consent. One observer, using a light microscope (IX70, Olympus Optical Co. Ltd, Tokyo, Japan) under 15–40X magnification, selected 60 teeth (52 central and lateral incisors and 8 canines) with no fractures on the external root surfaces. The teeth were stored in distilled water until the experimental procedures were completed. Digital radiographs were made in the buccolingual and mesiodistal directions. On the basis of root length, mesiodistal and buccolingual dimensions, 60 teeth were allocated into three identical experimental groups (*n* = 15) and one control group (*n* = 15).

The teeth in the control group (Group A) were left unprepared. In the other three groups, a size 10 K-file (Mani Inc., Tochigi-Ken, Japan) was placed passively in each root canal until it reached the apical foramen. The working length was 0.5 mm shorter than the measured length. The root canals were considered to be narrow, as the size 10 K-file did not easily access the working length. The teeth were mounted vertically in self-cured acrylic (Orthoplast; Vertex, Zeist, Netherlands) blocks. Blocks were placed in a positioning jig that allowed for resin block stabilization during preparation.

In group B, the canals were instrumented with stainless steel K-files (Mani Inc., Tochigi-Ken, Japan) by the step-back technique. Size 10–25 K-files were used up to the full working length, to constitute apical preparation. The mid-root and coronal parts of the canals were also prepared by the step-back technique, but with size 30 increased to size 70 K-files, whilst the working length (1 mm) was decreased with each instrument change. Coronal enlargement was carried out with Gates-Glidden drills 1 (size #50), 2 (size #70) and 3 (size #90).

In groups C and D, root canals were prepared with WaveOne® Primary instruments and RECIPROC® R25 instruments, respectively. WaveOne® and RECIPROC® instruments were used with a reciprocating gentle in-and-out motion powered by a torque-limited electric motor (Silver RECIPROC® motor,VDW, Munich, Germany) according to the manufacturer's recommended settings.

In group C, the WaveOne® Primary instrument (tip size ISO 25 with a 0.08 taper in the apical 3 mm with a subsequent decreasing and variable taper) was used in the ‘WAVEONE ALL’ mode at a speed of 350 rpm. The flutes of the instrument were cleaned after three in-and-out movements.

In group D, the RECIPROC® R25 instrument (tip size ISO 25 with a 0.08 taper in the apical 3 mm with a subsequent decreasing and variable taper) was used with the ‘RECIPROC ALL’ mode at a speed of 300 rpm. The flutes of the instrument were cleaned after three in-and-out movements.

A single operator (an endodontist previously trained in the use of the reciprocating instrument) performed all root canal preparations. A 1 mL quantity of 5.25% sodium hypochlorite was used after each change of instrument or every three in-and-out motions of reciprocating instruments. After canal preparation, 5 mL quantity of 17% ethylenediaminetetraacetic acid (EDTA) was added followed by a final rinse in distilled water. Root canals were irrigated by means of a 30-gauge NaviTip irrigation needle (Ultradent, South Jordan, UT, USA) placed as deeply as possible in the canal without binding.

Each instrument was used once. The apical preparation was done up to size ISO 25 in all experimental groups. Teeth were again stored in distilled water until being sectioned.

### Sectioning and observation of roots

**Figure 1.**
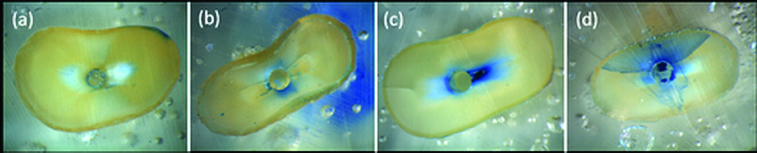
Representative microscopic image showing (a) ‘no defect’ at 3 mm level, (b) ‘partial crack’ at 3 mm level, (c) ‘craze line’ at 6 mm level and (d) ‘fracture’ at 3 mm level.

Resin blocks were placed on an Isomet 1000 precision saw (Buehler, Lake Bluff, IL, USA) equipped with a 0.3 mm diamond disc (Buehler) and water cooling. Horizontal sections were made at 3, 6 and 9 mm from the apex. Samples were stained with methylene blue and viewed through a stereomicroscope (Leica MZ75, Leica Imaging Systems Ltd, Cambridge, England) at a magnification of 24X for the 3 mm apical slices and 18.75X for the rest. All slices were then photographed with a digital camera (Olympus x-835, Olympus Co. Ltd, Tokyo, Japan) equipped with appropriate software (Leica QWin Leica Imaging Systems Ltd, Cambridge, England). Two endodontists who were blind in respect of the experimental groups were calibrated based on the figures from [6] and assessed the images together. The dentinal defects and their locations were investigated in each horizontal section. ‘No defect’ was defined as root dentine without fracture or any other defect (Figure 1(a)). ‘Other defect’ was defined as partial cracks (extending from the canal walls into the dentine without reaching the outer surface) (Figure 1(b)) or craze lines (extending from the outer surface into the dentine but not reaching the canal lumen) (Figure 1(c)) extending from the outer surface of the root or inner canal wall.[6] ‘Fracture’ was defined as a crack extending from the canal lumen to the external surface of the root (Figure 1(d)).

### Statistical analysis

Data were analysed statistically by Fisher's exact and chi-square tests at a significance level of *p* < 0.05.

## Results and discussion

**Figure 2. f0002:**
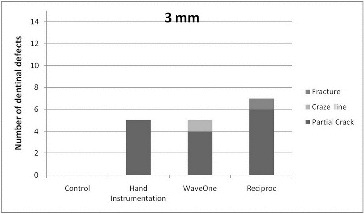
Distribution of number of dentinal defects at 3 mm root level (more than one defect per slice was possible).

**Figure 3. f0003:**
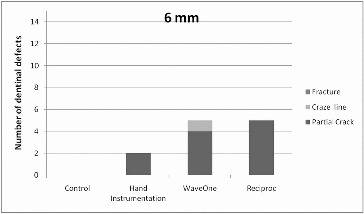
Distribution of number of dentinal defects at 6 mm root level (more than one defect per slice was possible).

**Figure 4.**
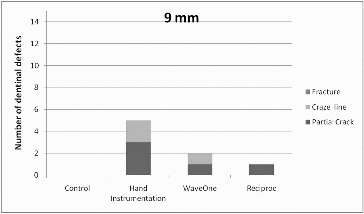
Distribution of number of dentinal defects at 9 mm root level (more than one defect per slice was possible).

Figures 2– 4 summarize the results at three levels (3, 6 and 9 mm). No defects were observed in the unprepared group. Fracture of the root dentine was observed only in the RECIPROC® group at the 3 mm level. All instruments caused dentinal defects, with no significant differences between the instrument systems.[Fig f0002] [Fig f0003]

All experimental groups demonstrated significantly more defects at the 3 mm level when compared with the unprepared group (chi-square test, *p* = 0.032). At the other levels, the difference between experimental groups and the control group in the appearance of all defects was not significant (Table 1).

**Table 1.** Numbers of teeth presenting dentinal defects in different cross-sectional slices.

	Root levels		
Groups	3 mm dentinal defect	6 mm dentinal defect	9 mm dentinal defect
Hand instrumentation *n* = 15	5	2	4
WaveOne® *n* = 15	5	5	2
RECIPROC® *n* = 15	7	5	1
Control	0	0	0
*P* value	0.032*	0.058	0.129

*p < 0.05

When all groups with the overall appearance of defects were considered, the 3 mm level presented significantly more defects than the 9 mm level (chi-square test, *p* = 0.022). However, there was no significant difference between the other levels.

VRF is a serious clinical concern during root canal treatment, compromising the prognosis of many cases in which extraction is the only possible treatment option. Morfis [19] reported that VRFs attributed to endodontic procedures were found in 3.69% of endodontically treated teeth. VRFs may originate from stress concentrations arising from mechanical preparation.[9] Instrumentation may also weaken the root structure and increase its susceptibility to defects such as craze lines (lines on the root surface which do not extend toward the root canals) or incomplete cracks (cracks which start in the canal lumen and do not reach the outer surface).[6] These dentinal defects may possibly lead to VRFs during root canal filling, retreatment and post placement, or simply because of masticatory forces.[20] In this study, dentinal defects generated by different instruments were directly observed, and the presence of dentinal defects and VRFs in root dentine was evaluated.

Unfortunately, excessive force during tooth extraction may create crack lines. Hence, all teeth were observed under a microscope so that teeth without defects would be selected. Tooth-sectioning may also generate dentinal defects. However, previous studies that used the sectioning method did not report crack formation in unprepared groups.[6,7,12,18] Similarly, the control group showed no defects, ensuring that any dentinal defects detected subsequently occurred during the instrumentation procedures.

Root and canal morphology are the main factors in VRF formation.[21] Roots that are wide buccally and lingually, but thinner mesially and distally, tend to fracture more often.[22] A finite-element analysis study showed that stress concentrations were highest in oval roots, and greater tensile stress was observed in the buccolingual direction as proximal dentine thickness was reduced.[3] Mandibular incisors, having generally oval inner and outer root shapes and thin mesiodistal dentine walls, are more prone to VRFs. Mandibular incisors were used in this study. Our results showed that more defects were observed in apical sections of the roots when compared with coronal sections. Although the oval root canals tapered to rounder shapes apically,[23] the thinner dentine walls of the apical root could have possibly induced more crack formation. Small WaveOne® instruments would be adequate for the narrow canals of mandibular anterior teeth. However, WaveOne® primary instruments and the RECIPROC® R25 instrument, both of which have a tip size of 0.25 mm, were used in this study, to provide a standardized research method.

Only one fracture was observed in this study, confirming that fractures do not occur immediately after root canal preparation. Although the sample size was small, this finding was corroborated in a similar study with a much larger sample size. Bier et al. [12] found no complete fracture in any of the samples prepared with either hand or rotary instruments. However, dentinal defects like craze lines or incomplete cracks may turn into fractures over time. Thus, their potential effect on root fracture makes these defects clinically significant. Conversely, there is no study focusing on the correlation between the results revealed in this kind of *in vitro* study and clinical outcome. Unfortunately, the *in vitro* conditions of the study limit clinical relevance, due to variability in study design and evaluation techniques. Although, in this study, it was preferred that an entire tooth be prepared, instead of a root, to mimic the clinical situation, the absence of natural PDL (periodontal ligament) was a significant limitation. Embedding the teeth directly in the resin blocks would have led to additional stabilization of the tooth structure. However, there is no consistent and standard experimental design for PDL simulation. Furthermore, various materials have been used to mimic stress distribution mechanisms.[5,10,24,25] Soros et al. [26] stated that elastomeric materials are insufficient to represent exactly both the natural PDL and what may be possible in a clinical situation. There are some studies which have used PDL simulation in experimental steps performed after root canal preparation. In one such study, the roots were instrumented without PDL simulation and subsequently coated by an impression material to provide better stress distribution against lateral forces during filling procedures.[6] Barreto et al. [27] attempted to use PDL simulation after preparation and filling procedures, just before the mechanical cycling experiment, to maintain force distribution during fatigue loading. However, some studies did not use PDL simulation in any experimental stage.[12,18]

There are several studies claiming that hand instruments did not damage the root canal wall.[10,12,28] This was attributed to the removal of less dentine as a consequence of less taper of the hand instruments.[6,12] In this study, the hand instrument group showed only partial cracks at the 3- and 6-mm levels. Moreover, there were fewer or equal numbers of dentinal defects compared with those in the reciprocating instrument groups. However, this was just the opposite when 9-mm sections were observed. The hand instrument group presented a higher number of dentinal defects, which were not just partial cracks but also craze lines. Increased numbers of coronally located cracks might indicate the possibility of excessive tapering of narrow root canals with Gates Glidden drills (#90) at the coronal third. Gates Glidden drills have been found to have the potential to reduce residual dentinal thickness.[29,30] This excessive reduction of hard tissues guaranteed on the one hand the same preparation size compared to the reciprocating single instruments but weakened the roots on the other hand making them more prone to fracture.

NiTi rotary instruments create smooth, round root canals and more controlled tapers than hand instruments. Thus, they were expected to produce uniform stress distribution and less overall stress on the canal walls.[9,31] However, the root canals prepared with NiTi rotary instruments were found to have a higher incidence of dentinal defects compared with those prepared by hand instruments, and this was attributed to greater taper and a higher number of rotations with rotary instruments.[12] This finding did not support those from the previous study, which stated that increased apical enlargement and increased taper did not weaken roots any more than hand instruments and might even increase fracture resistance.[32] In an earlier study, Wilcox et al. [5] considered the roots more susceptible to fracture when more dentine was removed. Sathorn et al. [21] was in agreement with this study; however, they added that a reduction of dentine was only one of many factors. Together with dentine reduction, parameters such as curvature of the external proximal root surface and canal size and shape might interact in influencing fracture susceptibility and pattern.[21] Kim et al. [33] evaluated the potential relationship between the design of NiTi instruments with different shaft geometries and VRFs and attributed dentinal defects to the instrument design affecting apical stress and strain concentrations during instrumentation. RECIPROC® and WaveOne® instruments are both made of a special NiTi alloy called M-wire, having the advantages of increased flexibility and resistance to cyclic fatigue. However, they differ in tip and taper designs. RECIPROC® has an S-shaped cross-sectional design with sharp cutting blades, whereas WaveOne® has a convex triangular cross-section from D9 to D16 and modified convex triangular cross-section from D1 to D8 with a non-cutting modified guiding tip.[34] Kim et al. [14] reported that WaveOne® showed higher torsional resistance than ProTaper and RECIPROC® instruments. This was related to the large cross-sectional area of WaveOne® and the mechanical characteristics of the NiTi alloy. In this study, no significant difference was found between the two reciprocating instruments and hand instruments in all root sections. The reciprocating motion might minimize the torsional stresses and reduce the screwing effect of the instrument,[16,17] because clockwise and counterclockwise rotations allow the instrument to cut and consecutively disengage dentine. Future studies with more focus on the stresses created by reciprocating motion in root dentine are therefore suggested.

A previously published study reported that RECIPROC® instruments were associated with more complete cracks compared with rotary instruments.[18] However, the observed difference between RECIPROC® and WaveOne® was not significant.

According to the results of this study, all instruments, including hand instruments, caused dentinal defects. In accordance with the results of Burklein et al. [18], RECIPROC® and WaveOne® produced similar numbers of dentinal defects with no significant difference. The lack of significance may be attributable to the small sample size. Another possible explanation for this finding might be that both instruments are used in a reciprocating motion and are made of the same material. It is therefore presumed that the difference in instrument design did not influence the formation of cracks.

## Conclusion

Within the limitations of this *in vitro* study, it may be concluded that the use of hand or reciprocating instruments could induce the formation of dentinal defects during root canal preparation.

## Disclosure statement

The authors deny any conflicts of interest.
